# Metabolomic Approaches to Study the Potential Inhibitory Effects of Plantaricin Q7 against *Listeria monocytogenes* Biofilm

**DOI:** 10.3390/foods13162573

**Published:** 2024-08-17

**Authors:** Yinxue Liu, Yisuo Liu, Linlin Hao, Jiayuan Cao, Lu Jiang, Huaxi Yi

**Affiliations:** College of Food Science and Engineering, Ocean University of China, Qingdao 266000, China; liuyinxue_1121@126.com (Y.L.); liuyisuo@stu.ouc.edu.cn (Y.L.); haoqh5la@163.com (L.H.); jiayuanalice@163.com (J.C.); lujiang0806@163.com (L.J.)

**Keywords:** plantaricin Q7, *Listeria monocytogenes* biofilm, metabolomics, inhibition, reduction

## Abstract

*Listeria monocytogenes* is a serious pathogen and can exacerbate harmful effects through the formation of biofilm. Inhibition of or reduction in *L. monocytogenes* biofilm is a promising strategy to control *L. monocytogenes* in the food industry. In our previous study, it was found that plantaricin Q7 produced by *Lactiplantibacillus plantarum* Q7 could inhibit and reduce *L. monocytogenes* biofilm, but the specific mechanism remains unclear. In this study, the inhibitive and reduced activity of plantaricin Q7 on *L. monocytogenes* biofilm was investigated by metabolomics. The results showed that plantaricin Q7 inhibited the synthesis of *L. monocytogenes* biofilm mainly through purine metabolism and glycerol phospholipid metabolism, and the key differential metabolites included acetylcholine and hypoxanthine with a decrease in abundance from 5.80 to 4.85. In addition, plantaricin Q7 reduced the formed *L. monocytogenes* biofilm by purine metabolism and arginine biosynthesis, and the main differential metabolites were N-acetylglutamate and D-ribose-1-phosphate with a decrease in abundance from 6.21 to 4.73. It was the first report that purine metabolism and amino acid metabolism were the common metabolic pathway for plantaricin Q7 to inhibit and reduce *L. monocytogenes* biofilm, which could be potential targets to control *L. monocytogenes* biofilm. A putative metabolic pathway for *L. monocytogenes* biofilm inhibition and reduction by plantaricin Q7 was proposed. These findings provided a novel strategy to control *L. monocytogenes* biofilm in food processing.

## 1. Introduction

*L. monocytogenes* is one of the serious virulent psychrophilic bacteria [1]. Food is the main carrier of *L. monocytogenes*, such as meat, eggs, aquatic products, dairy products and vegetables [2]. Despite significant efforts to combat *L. monocytogenes* contamination, *L. monocytogenes* continues to pose a major challenge to the food industry due to its wide-ranging presence and persistence in manufacturing processes. *L. monocytogenes* is the etiological agent of listeriosis, one of the deadliest foodborne diseases in industrialized countries [3]. Infectious diseases caused by *L. monocytogenes* have become a serious public health problem due to its high fatality rate, especially for those with low immunity (the elderly, pregnant women and newborns) and immune deficiency [4]. In the United States, approximately 20–65% of deaths from foodborne infections are caused by *L. monocytogenes* [5].

Biofilms are special microbial communities produced by planktonic cells, which mainly included extracellular polysaccharides, nucleic acids and proteins [6]. On the one hand, *L. monocytogenes* biofilm can form on the surfaces of various medical devices and food-processing machines, bringing new challenges for pharmaceutical and food fields. Cross-contamination caused by *L. monocytogenes* biofilm can easily occur during food processing, transportation, production and storage [7]. On the other hand, the biofilm state of *L. monocytogenes* can withstand more severe conditions such as high temperature, high salt, dryness, disinfectants, and UV light. Elimination of *L. monocytogenes* biofilm usually requires higher doses of antimicrobial drugs than planktonic *L. monocytogenes* [8]. Therefore, *L. monocytogenes* biofilm has been an important issue in food safety. However, the conventional disinfection and sterilization methods cannot completely remove the formed *L. monocytogenes* biofilm. There is an urgent need to develop a method that can effectively kill *L. monocytogenes* in the biofilm state.

Bacteriocins are ribosomally synthesized antibacterial peptides in many bacterial species which have been suggested as a potential substitute for chemical preservatives and antibiotics [9]. In our previous study, *L. plantarum* Q7 was proven to have broad-spectrum antibacterial activity, especially against *L. monocytogenes* [10]. Then, by excluding organic acids, hydrogen peroxide experiments, and papain, bromelain, trypsin, pepsin and proteinase K decomposition experiments, we determined that the antibacterial effect of *L. plantarum* Q7 was achieved by plantaricin Q7 [11]. In addition, we isolated and purified plantaricin Q7 by ultrafiltration concentration and filtration chromatography [12].

Further, it was found that plantaricin Q7 not only inhibited the formation of *L. monocytogenes* biofilm but also reduced the mature *L. monocytogenes* biofilm [13]; however, the mechanism is unclear. Biofilm formation is closely related to bacterial metabolism. In recent years, metabolomics has become an important tool in food science research, especially in solving problems related to food safety, quality and nutrition. Favre et al. [14] used LC-MS metabolomics to identify four species of bacteria and culture parameters that form marine biofilms. Munusamy et al. [15] analyzed the different metabolic processes of *Candida albicans* SC5314 under the modes of planktonic cells and biofilm cells. Guo and Lu [16] explored the regulatory role of Mn^2+^ on biofilm formation in *Escherichia coli* by precision-targeted metabolomics. In this study, the inhibition and reduction in activity of plantaricin Q7 on *L. monocytogenes* biofilm were investigated by metabolomics, and the possible metabolic pathway was postulated, which laid the foundation for the development of plantaricin Q7 as a food preservative to control *L. monocytogenes* biofilm.

## 2. Materials and Methods

### 2.1. Strain Culture and Preparation of Plantaricin Q7

*L. plantarum* Q7 was isolated from yak milk Qinghai, China, cultured into De Man, Rogosa and Sharpe (MRS) broth (Qingdao Hopebio Technology, Qingdao, China) and stored in the Functional Dairy and Probiotic Engineering Laboratory (Ocean University of China, Qingdao, China). *L. monocytogenes* ATCC 19115 as the indicator strain was obtained from the Food Safety Laboratory (Ocean University of China, Qingdao, China) and cultured in Brain–Heart Infusion (BHI) broth (Qingdao Hopebio Technology, China). The strains were inoculated in broth at the concentration of 2% (*v*/*v*) and activated twice at 37 °C for 24 h [11]. Plantaricin Q7 was prepared and determined as follows: after centrifugation of the fermentation broth of *L. plantarum* Q7 at 2000× *g* for 10 min, the supernatant was adjusted to pH 6.0 with 1 M NaOH and filtered through a 0.22 µm filter. Then, a 3 KDa ultrafiltration tube was used for ultrafiltration at 2000 g for 30 min at 4 °C. After ultrafiltration, the protein concentration of plantaricin Q7 was determined by microplate BCA protein detection kit (Thermo Fisher Scientific, Waltham, MA, USA).

### 2.2. Determination of the Antimicrobial Activity of Plantaricin Q7

According to the method of Dosler et al. [17], minimum inhibitory concentration (MIC), minimum bactericidal concentration (MBC) and minimum biofilm eradication concentration (MBEC) were determined by serial two-fold dilution (15,840, 7920, 3960, 1980, 990, 495, 248, 124, 62, 31, 16, 8, 4, 2, 0 μg/mL) of plantaricin Q7 in 96-well plates (Costar, Corning, NY, USA). Briefly, by using nisin (Sigma-Aldrich, MO, USA) as positive control and phosphate-buffered saline (PBS, Solarbio, Beijing, China) solution as negative control, the MIC defined as the minimum plantaricin Q7 concentration that completely inhibited the visible growth of *L. monocytogenes* was assayed. All solutions in the MIC, 2× MIC, 3× MIC wells were selected for coating on BHI medium, and MBC was defined as the lowest concentration of plantaricin Q7 that killed at least 99.9% of the initial inoculums after counting. The formation of biofilms was as follows: 200 μL *L. monocytogenes* solution was added to a 96-well plate and the bacterial solution was removed after 24 h culture. Then, the remaining planktonic *L. monocytogenes* was removed by washing three times with PBS solution to obtain the mature biofilm. Continuously, diluted plantaricin Q7 was absorbed into the corresponding pore and then cultured at 37 °C for 24 h. After culture, the plate was washed twice with sterile PBS solution. The pore contents were removed and placed in 1 mL PBS solution, then ultrasounded in a sonicating water bath (Taisite, Tianjin, China) for 10 min to destroy the biofilm, and 100 μL samples were cultured on BHI. Colonies were counted after 24 h at 37 °C. MBEC was defined as the lowest plantaricin Q7 concentration that prevented bacterial regeneration in biofilm.

### 2.3. Metabolomic Sample Preparation

*L. monocytogenes* biofilm was established in accordance with previous methods [18]. For the inhibition of *L. monocytogenes* biofilm formation by plantaricin Q7, *L. monocytogenes* was incubated for 8 h and diluted to 10^7^ CFU/mL with BHI; then, 5 mL of bacterial suspension and the final concentration of 7920 μg/mL of plantaricin Q7 were injected into each well of a 6-well cell culture plate (Costar, Corning, NY, USA) with a glass coverslip (25 mm × 25 mm), and finally incubated for 24 h at 37 °C. The group without plantaricin Q7 was used as the control. For the reduction in *L. monocytogenes* biofilm by plantaricin Q7, 5 mL suspension of *L. monocytogenes* with a concentration of 10^7^ CFU/mL was added to a 6-well cell culture plate containing the glass coverslips for 24 h to form a mature biofilm; then, the biofilm-formed glass coverslips were gently transferred with a sterile needle and tweezers to a new 6-well cell culture plate containing 7920 μg/mL plantaricin Q7. Plantaricin Q7 was replaced with PBS as the control. After reaching the indicated incubation time, the biofilm was gently washed 3 times with sterile PBS, and collected using a cell scraper (Thermo Fisher Scientific, MA, USA) and transferred into 1.5 mL tubes. All the samples were immediately washed three times with sterile PBS and centrifuged at 2000× *g* for 10 min at 4 °C. The cell precipitates were frozen with liquid nitrogen for 15 min and stored at −80 °C [19]. All experiments were repeated three times.

### 2.4. Metabolite Extraction

Into the sample tube, sterile, enzyme-free zirconia grinding beads with a diameter of 4 mm (Servicebio, Wuhan, China) and 400 µL of extraction solution (methanol/water = 4:1 (*v*/*v*)) (Fisher Chemical, Waltham, MA, USA) containing 0.02 mg/mL L-2-chlorophenylalanine (Adamas-beta, Shanghai, China) were added. Then, the samples were ground at −10 °C for 5 min (50 Hz) using a frozen tissue grinder (Wonbio, Shanghai, China), extracted by ultrasound at 5 °C for 30 min (40 kHz, 360 W, Scientz, Ningbo, China), and placed at −20 °C for 30 min, followed by centrifuging at 13,000× *g* under 4 °C, for 15 min. Finally, the supernatants were transferred to vials for LC-MS/MS analysis.

### 2.5. LC-MS/MS Analysis

Metabolite samples were loaded onto an ACQUITY UPLC HSS T3 column (100 mm × 2.1 mm i.d., 1.8 µm; Waters, Milford, CT, USA). The mobile phase A consisted of 95% water and 5% acetonitrile (containing 0.1% formic acid), and mobile phase B consisted of 47.5% acetonitrile + 47.5% isopropanol + 5% water (containing 0.1% formic acid). The flow rate was 0.4 mL/min for equilibrating the systems, and the detailed parameters of the mobile phase elution gradient are shown in Table S1. The sample injection volume was 2 µL and the column temperature was maintained at 40 °C. All these samples were stored at 4 °C for the duration of the analysis. The mass spectrometric data were collected using a Thermo UHPLC-Q Exactive Mass Spectrometer (Thermo Fisher Scientific, MA, USA) equipped with an electrospray ionization (ESI) source. The mass spectral signals were acquired in positive and negative ion scanning modes. The specific ESI source parameters were set as follows: sheath gas flow rate, 50 psi; aux gas flow rate, 13 psi; heater temperature, 425 °C; capillary temperature, 325 °C; ion-spray voltage floating (ESI+), +3500 V; ion-spray voltage floating (ESI−), −3500 V; normalized collision energy, 20–40–60 eV. The detection was carried out over a mass range of 70–1050 mass-to-charge ratio (*m*/*z*).

### 2.6. Metabolite Identification

Progenesis QI (v2.0, Waters, Milford, CT, USA) software was used to identify the characteristic peak search library, and the MS/MS mass spectrometry information was matched with the metabolic database. The MS mass error was set to <10 ppm, and the metabolites were identified according to the secondary mass spectrometry matching score. Fragmentation score and theoretical fragmentation methods were used to control false positive results and qualitative accuracy.

### 2.7. Statistical Analysis

Multivariate statistical analysis of the collated data was carried out using SIMCA software (v14.1). The test samples were divided into two groups, Inhibition_TR vs. Inhibition_Control, and Reduction_TR vs. Reduction_Control. Principal component analysis (PCA) and partial least squares discriminant analysis (PLS-DA) were performed to confirm the degree of data dispersion. The differential metabolites of the inhibition groups and the reduction groups were screened according to the criteria of the variable importance in the projection VIP > 1 and *p* value < 0.05. The Benjamini–Hochberg method was used to correct the *p* value, and then mapped into their biochemical pathways based on the Kyoto Encyclopedia of Genes and Genomes (KEGG, http://www.genome.jp/kegg/, accessed on 5 February 2024). All experiments were repeated three times, and the data are shown as mean ± standard deviation (SD).

## 3. Results and Discussion

### 3.1. Determination of Plantaricin Q7 Antimicrobial Activity

In our previous research, the results showed that there was no significant difference in the antibacterial activity of *L. plantarum* Q7 supernatant before and after excluding organic acid and adding catalase, illustrating that the bacteriostatic characteristic of *L. plantarum* Q7 strains was not attributed to organic acids and hydrogen peroxide. In addition, the incubation of supernatants with pepsin led to the loss of antibacterial ability of *L. plantarum* Q7, which demonstrated that the antibacterial products of *L. plantarum* Q7 might be polypeptides or proteins. Therefore, the antibacterial effect of *L. plantarum* Q7 was achieved by the secretion of plantaricin Q7 [11].

Nisin was used widely as food preservatives. In our previous study, we compared the inhibitory effect of nisin and plantaricin Q7 on *L. monocytogenes*. It was found that plantaricin Q7 could cause cell membrane disruption of *L. monocytogenes* within 20 min, whereas nisin showed no effect [12]. The MIC, MBC and MBEC of plantaricin Q7 on *L. monocytogenes* were determined as shown in Figures S1 and S2, and Tables S2–S5 with nisin as positive control and PBS as negative control. The MIC of plantaricin Q7 against *L. monocytogenes* was determined to be 495 μg/mL, and the MBC and MBEC of plantaricin Q7 to *L. monocytogenes* were 990 μg/mL and 7920 μg/mL, respectively. The concentrations were similar to that of nisin in inhibiting *L. monocytogenes* reported by Yan et al. (2024) [20], and the MBEC of plantaricin Q7 was used to inhibit and reduce *L. monocytogenes* biofilm in this study.

### 3.2. Metabolomic Samples’ Statistical Analysis

**Figure 1:**
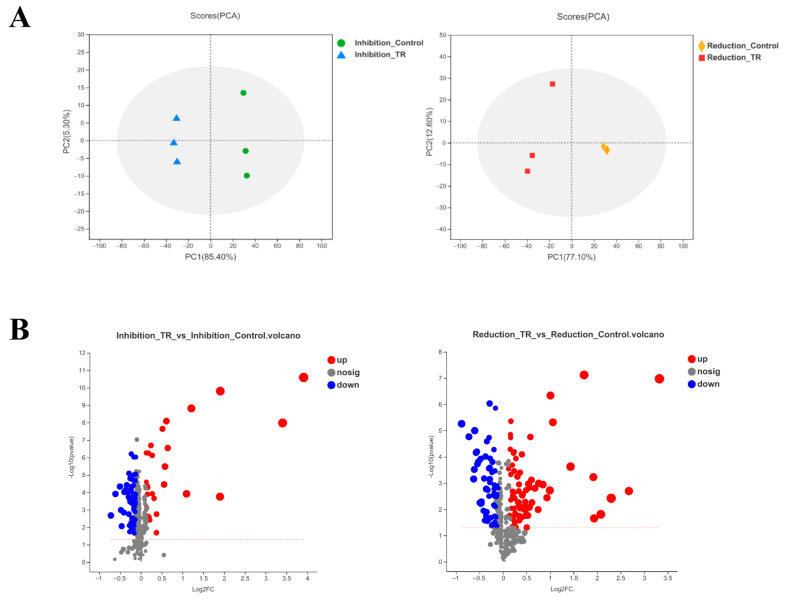
PCA score plots and PLS-DA score plots. (**A**) PCA scores of samples in inhibition group and reduction group. (**B**) Volcano plot of inhibition group and reduction group. The points below the red dashed line indicate no change.

The samples were determined by UHPLC-Q Exactive HF-X system. In positive-ion mode, a total of 430 compounds were detected in the inhibition group and 423 compounds were detected in the reduction group. In negative-ion mode, 219 compounds were identified in the inhibition group and 213 compounds were identified in the reduction group. The total ion chromatograms in positive-ion mode are shown in Figure S3, which showed that there were certain differences in the number of chromatographic peaks, ionic strength and retention time in different groups. For multivariate analysis, PCA score plots showed a trend towards separation between samples, with metabolic differences between groups (Figure 1A). It was shown that the clusters between groups were separated significantly in the PLS-DA score plot (Figure S4). The validation of the PLS-DA models was carried out using the permutation tests, and the evaluating parameters from PLS-DA model were R^2^Y = 0.998, Q^2^ = 0.996 (Inhibition_TR vs. Inhibition_Control), and R^2^Y = 0.965, Q^2^ = 0.940 (Reduction_TR vs. Reduction_Control), which suggested that the model had a good degree of discrimination and prediction.

Quantification of metabolite levels was based on the area of the metabolite peaks in the mass spectrograms. In the Inhibition_TR vs. Inhibition_Control group, 91 significantly different metabolites were detected, of which 29 metabolites were up-regulated and 62 metabolites were down-regulated. In total, 132 significantly different metabolites were detected in the group of Reduction_TR vs. Reduction_Control, and it was found that 75 metabolites were up-regulated and 57 metabolites were down-regulated. The volcano map could visualize the screened differential metabolites. As shown in Figure 1B, each point represents a metabolite, and larger scatter points indicate larger VIP values in the PLS-DA model. The up-regulation or down-regulation of metabolites might play a key role in the formation of *L. monocytogenes* biofilm, which led to the performance of further analysis.

### 3.3. Differential Metabolite Classification

**Figure 2:**
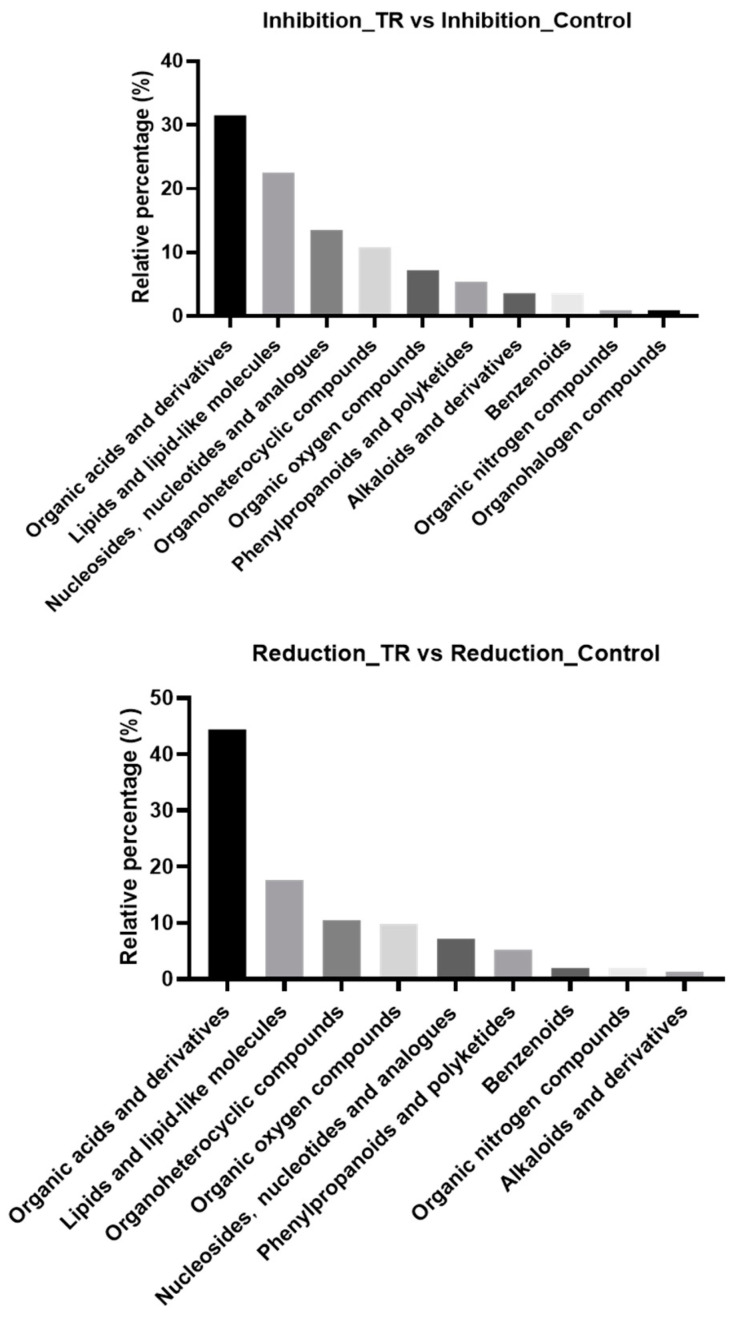
Differential metabolite classification in inhibition group and reduction group.

It was reported that organic acids, lipids and nucleotides were key substances associated with the formation of *Bacillus licheniformis* biofilm and *Cryptococcus neoformans* biofilm [21,22]. However, the biofilm-formation-associated metabolites of *L. monocytogenes* have not yet been reported. In this study, the differential metabolites of *L. monocytogenes* biofilm treated with plantaricin Q7 were classified as shown in Figure 2. In the inhibition groups, the differential metabolites were divided into the following categories: organic acids and derivatives, lipids and lipid molecules, nucleotides and analogues, and organic heterocyclic compounds and organic oxygen compounds, and the percentages were 31.53%, 22.52%, 35.51%, 10.81% and 7.21%, respectively. In the reduction groups, the differential metabolites were divided into organic acids and derivatives, lipids and lipid molecules, organic heterocyclic compounds, organic oxygen compounds, and nucleotides and analogues, and the percentages were 44.44%, 17.65%, 10.46%, 9.80% and 7.19%, respectively. These results indicated that *L. monocytogenes* biofilm treated with plantaricin Q7 had significant changes in the expression of metabolites, and the above compounds were particularly important for the formation of *L. monocytogenes* biofilm.

### 3.4. Metabolic Pathway Enrichment Analysis

**Figure 3:**
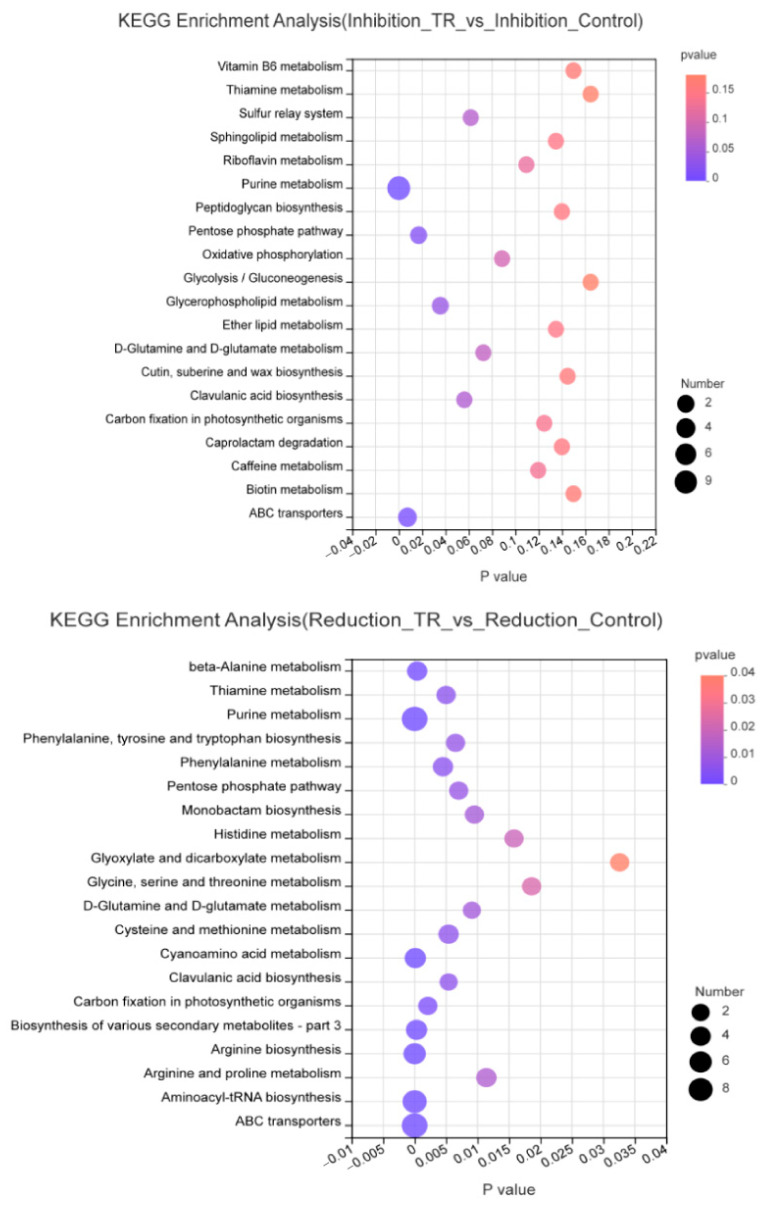
Enrichment analysis bubble diagrams of KEGG metabolic pathway in samples from inhibition group and reduction group.

**Figure 4:**
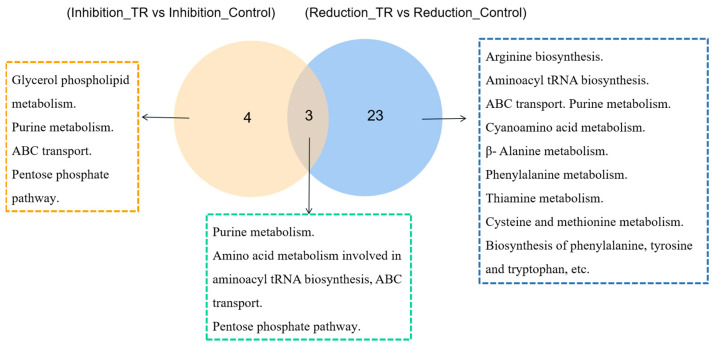
Venn diagram of enrichment pathway. The yellow circle represents the Inhibition_TR vs. Inhibition_Control group, and the blue circle represents the Reduction_TR vs. Reduction_Control.

KEGG enrichment pathways were calculated based on different expressed metabolites, and the pathway was considered to be significantly enriched when the corrected *p* value was <0.05. The results are presented as bubble maps (Figure 3) and a bar chart (Figure S5). Between Inhibition_TR vs. Inhibition_Control group, 32 metabolic pathways were identified, of which four pathways were significantly enriched with *p* < 0.05, including purine metabolism, ABC transport, pentose phosphate pathway and glycerol phospholipid metabolism. In total, 71 metabolic pathways were identified between Reduction_TR vs. Reduction_Control group, of which 23 pathways were significantly enriched with *p* < 0.05, including aminoacyl tRNA biosynthesis, purine metabolism, ABC transport, arginine biosynthesis, β-Alanine metabolism, and carbon fixation of photosynthetic organisms. It was speculated that significant metabolic pathways might be closely related to the activity of plantaricin Q7 inhibiting and reducing *L. monocytogenes* biofilm, which were enriched, and a Venn diagram was constructed (Figure 4). Among them, it was observed that the same pathways of two groups included purine metabolism, aminoacyl tRNA biosynthesis, ABC transport and other amino acid metabolism, and pentose phosphate pathway. The glycerol phospholipid metabolism pathway was more significant in the inhibition group, while the arginine biosynthesis pathway was more significant in the reduction group. These two metabolic pathways were the most enriched in metabolites. Wang et al. [21] found that amino acid metabolism was quite active during *B. licheniformis* biofilm formation and synergized by lipid metabolism, carbohydrate metabolism and nucleotide metabolism. In addition, Munusamy et al. [15] identified folate metabolism, fatty acid metabolism and amino acid biosynthesis as important pathways associated with biofilm synthesis in *C. albicans*. Biofilm formation in *Pseudomonas aeruginosa* was shown to be closely related to carbohydrate metabolism [23,24]. The up- and down-regulation of all the metabolites had important effects on those key pathways. The synergism and interaction from metabolism of lipids, amino acids, nucleotides and carbohydrates integrated the process of biofilm development of *L. monocytogenes*.

### 3.5. Effect of Plantaricin Q7 on Purine Metabolism and Biofilm Formation of L. monocytogenes

**Figure 5:**
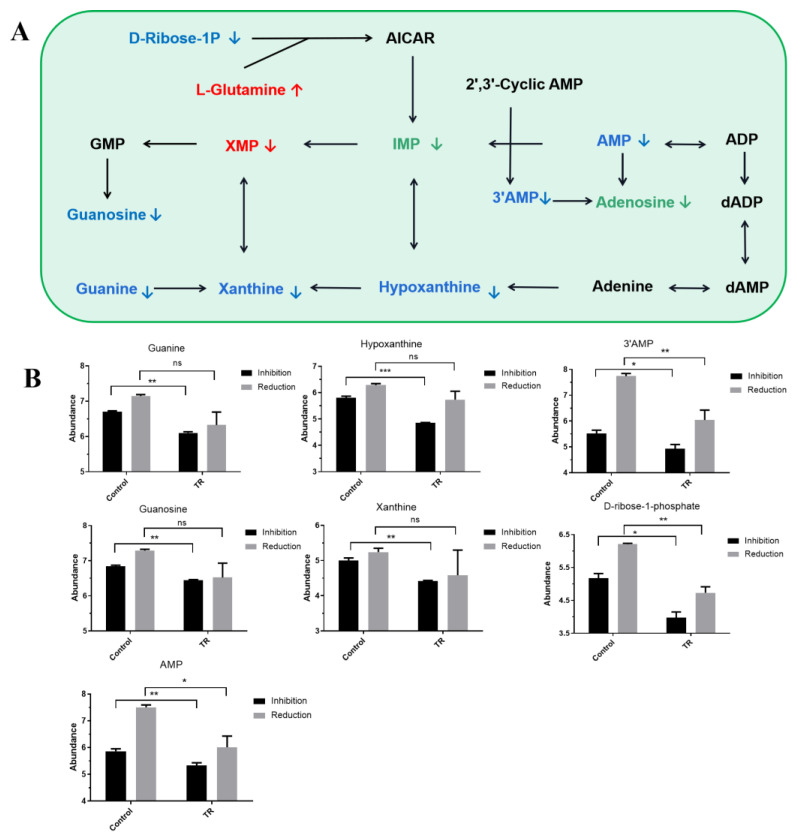
Purine metabolic pathway and major metabolites. (**A**) Purine metabolic pathway; (**B**) metabolites in purine metabolic pathway. Blue words indicate the metabolites with significant differences between the inhibition group and the reduction group, green words indicate the metabolites with significant differences only in the inhibition group, and red words indicate the metabolites with significant differences only in the reduction group. ↑ indicates up-regulation and ↓ indicates down-regulation. * *p* < 0.05, ** *p* < 0.01, *** *p* < 0.001, ns, not significant.

Based on the above pathway enrichment analysis, it was found that purine metabolism was the most common pathway for plantaricin Q7 to inhibit and reduce *L. monocytogenes* biofilms, and nine differential metabolites were detected in each group (Figure 5A). Among these differential metabolites, seven metabolites including guanine, hypoxanthine, 3′AMP, guanosine, xanthine, D-ribose-1-phosphate, AMP were down-regulated in both the inhibition and reduction groups (Figure 5B). Xanthine (the relative abundance decreased from 5.00 to 4.41 in the inhibition group and from 5.24 to 4.58 in the reduction group) was considered an important additive to bacterial cultures and could provide nutrients as a nitrogen source or substrate. In addition, it was shown that the down-regulation of hypoxanthine (the relative abundance decreased from 5.80 to 4.85 in the inhibition group and from 6.28 to 5.73 in the reduction group) was associated with the decline in flagellum motility of *L. monocytogenes*, which could indirectly affect the formation of *L. monocytogenes* biofilm [25]. Zhao et al. [26] found that the synthesis of hypoxanthine was inhibited after treatment of *Bacillus cereus* with plantaricin JLA-9, while other purine nucleotides used hypoxanthine as a synthesis precursor. Furthermore, it was reported that hypoxanthine positively regulated c-di-GMP synthesis, and a high level of c-di-GMP in cells could induce the synthesis of polysaccharide matrix and adhesin to facilitate the formation of biofilm, while a low level of c-di-GMP inhibited the synthesis of polysaccharide matrix and adhesin to slow down biofilm production in *P. aeruginosa* and *Vibrio parahaemolyticus* [23,27]. Therefore, it was suggested that plantaricin Q7 could inhibit and reduce *L. monocytogenes* biofilm by blocking the synthesis of purine metabolism pathway. Concretely, hypoxanthine was a key substance used for biofilm formation.

It is worth mentioning that the purine metabolism pathway was related to nucleic acid metabolism. Purines are products of the metabolism of nucleic acids (DNA and RNA) and are associated with biofilm formation. As shown in Figure 5B, the relative abundance of D-ribose-1-phosphate decreased from 5.17 to 3.97 in the inhibition group and 6.21 to 4.73 in the reduction group, and the relative abundance of AMP decreased from 5.86 to 5.33 in the inhibition group and 7.51 to 6.01 in the reduction group. The D-ribose-1-phosphate had a decisive influence on the generation of the entire purine metabolism. It was found that eDNA was one of the main components of biofilm matrix, which could assist in the progressive development of biofilms from the colony stage to maturity, and its interaction with extracellular polysaccharides facilitated the formation of more complex three-dimensional structures in biofilms [25]. Kang et al. [28] found that lactonic acid could inhibit DNA synthesis and the production of virulence factors, and ultimately lead to the reduction in methicillin-resistant Staphylococcus aureus biofilm. Thus, purine metabolism was deemed to play a crucial role in L. monocytogenes biofilm formation. In particular, the initial metabolite D-ribose-1-phosphate and the intermediate metabolite hypoxanthine were interrelated in purine metabolism, which could be directly related to *L. monocytogenes* biofilm formation.

### 3.6. Effect of Plantaricin Q7 on Amino Acid Metabolism and Biofilm Formation of L. monocytogenes

**Figure 6:**
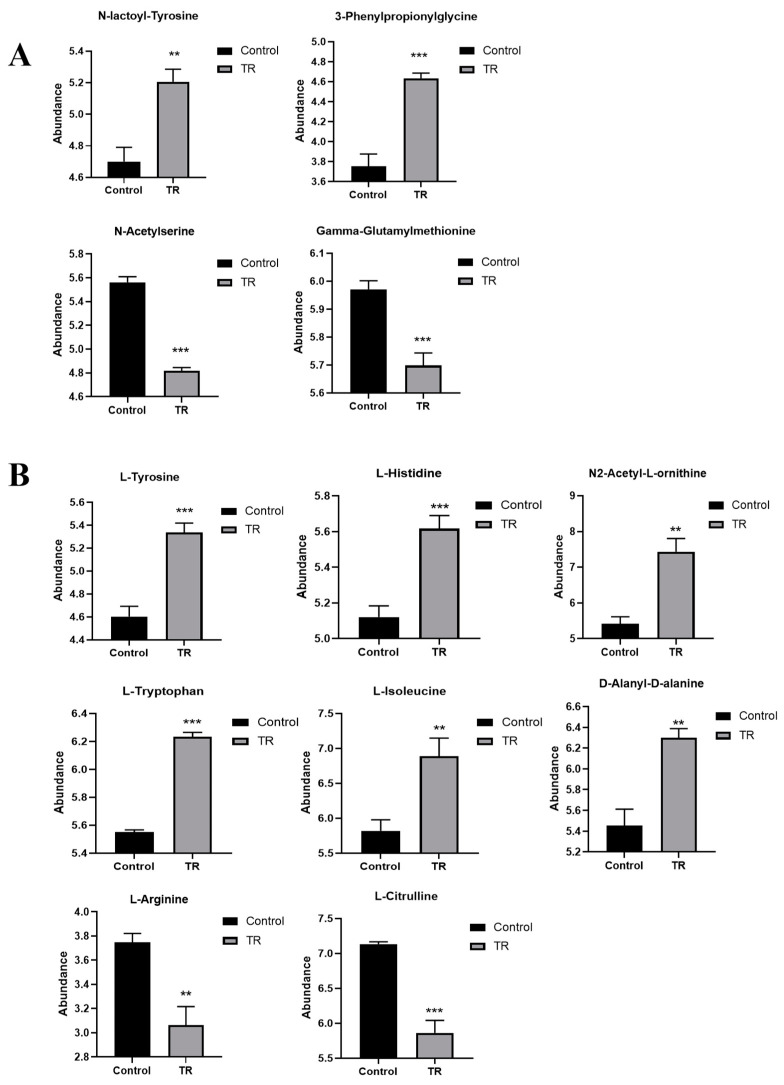
Effect of plantaricin Q7 on amino acid metabolism. (**A**) Inhibition_TR vs. Inhibition_Control group; (**B**) Reduction_TR vs. Reduction_Control group. ** *p* < 0.01, *** *p* < 0.001.

Amino acid metabolism was another significant pathway both in the inhibition and reduction in *L. monocytogenes* biofilm by plantaricin Q7. Amino acids were the material basis for the synthesis of enzymes and proteins in bacteria, and their levels had an impact on bacterial growth, cell wall and membrane synthesis, material transport and biofilm formation [29]. As shown in Figure 6, the differential metabolites of the inhibition group and the reduction group were screened according to the criteria of the variable importance in the projection VIP > 1 and *p* value < 0.05. In the inhibition group (Figure 6A), the up-regulated differential amino acids were N-lactoyl-tyrosine and 3-Phenylpropionylglycine, and the significantly down-regulated amino acids were N-acetylserine and gamma-glutamylmethionine. In the reduction group (Figure 6B), the significantly up-regulated amino acids included L-tyrosine, L-histidine, N2-acetyl-L-ornithine, L-tryptophan, L-isoleucine, D-alanyl-D-alanine, and the significantly down-regulated amino acids were L-arginine and L-citrulline. It was found that L-amino acids showed more changes in the reduction group than in the inhibition group, which suggested that L-amino acids contributed more to the formation of *L. monocytogenes* biofilm. It was reported that that L-aspartate and L-glutamine were associated with the two-component signal transduction system, which played a vital role in regulating bacterial biofilm formation, virulence gene expression, and cell wall synthesis [30,31]. Among the results of the reduction group (Figure 6B), the level of L-ornithine was increased, which indicated that it was involved in the reduction in *L. monocytogenes* biofilms. Miyamoto et al. [32] found that ornithine and alanine were involved in peptidoglycan and biofilm metabolism of hyperthermophile *Thermotoga maritima*, suggesting that these amino acids were necessary for hyperthermophile *T. maritima* to adapt to environmental changes. Conversely, some studies reported that D-amino acid slowed down the production of biofilm, such as *P. aeruginosa* biofilm [33], *S. aureus* biofilm [34], *Bacillus subtilis* biofilm [35], etc. In this study, D-alanine appeared only in the reduction group, indicating that D-alanine might participate in the reduction in mature *L. monocytogenes* biofilm. Consequently, the up- and down-regulation of amino acids metabolism might affect *L. monocytogenes* biofilm formation directly.

### 3.7. Effect of Plantaricin Q7 on Glycerol Phospholipid Metabolism and Biofilm Formation of L. monocytogenes

**Figure 7:**
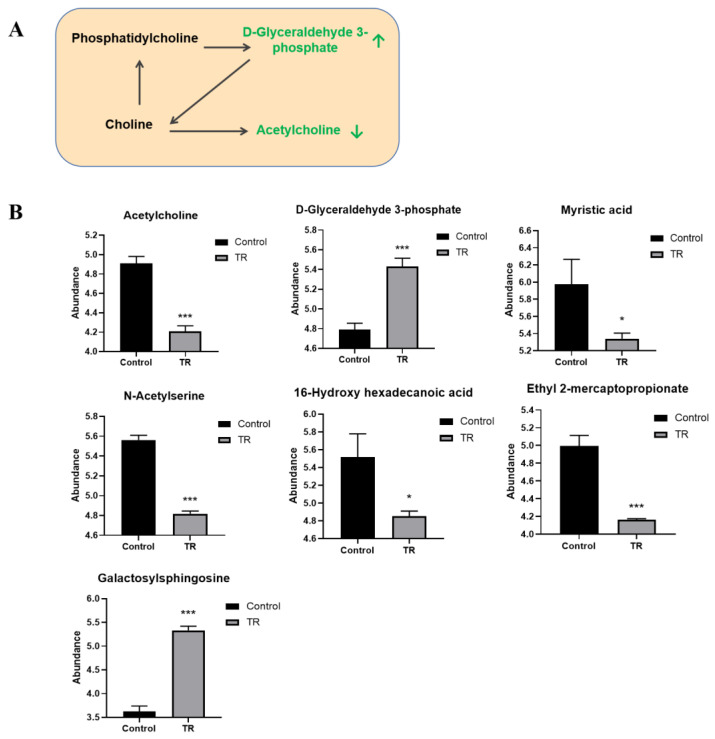
Glycerol phospholipid metabolic pathway and major metabolites. (**A**) Glycerol phospholipid metabolic pathway; (**B**) effect of plantaricin Q7 on metabolites of glycerol phospholipid metabolic pathway in the inhibition group. The black words in Figure 7A represent metabolites with no significant changes, while the green words represent metabolites with significant changes. Green ↑ indicates up-regulation and green ↓ indicates down-regulation. * *p* < 0.05, *** *p* < 0.001.

It was observed that glycerol phospholipid metabolism was significant in the inhibition group. Phospholipids are important components of cell membranes and play an important role of the energy metabolism of bacteria [21]. Changes in fatty acids indicated that the energy metabolism and the growth of *L. monocytogenes* was weakened by plantaricin Q7 treatment. Part of glycerophospholipid metabolic pathways and metabolites with significant differences were shown in Figure 7A,B. The contents of acetylcholine, D-glyceraldehyde 3-phosphate, myristic acid, N-acetylserine, 16-hydroxypalmitic acid, ethyl-2-mercaptopropionate and galactosylsphingosine were significantly changed after plantaricin Q7 treatment. Choline and phosphatidylcholine can produce acetylcholine and glycero-3-phosphocholine via intermediates. Interestingly, sphingomyelin has been reported to play an important role in the development of a variety of bacterial biofilms [36], and the important regulatory role of sphingosine on the formation of *Streptococcus mutans* biofilm has been demonstrated [37]. Similar results were reported in many bacterial species. It was found that the level of phospholipids in the biofilm was higher than that of planktonic cells [38]. Nwaiwu et al. [39] observed that phospholipids were essential for the formation of *L. monocytogenes* biofilm by using nuclear magnetic resonance (NMR) and energy dispersive X-ray spectroscopy (EDX) analysis. In addition, N-acetylserine and 16-hydroxypalmitic acid were intermediates of sphingolipid metabolism, and the down-regulation of these two metabolites might inhibit the formation of *L. monocytogenes* biofilm. The specific relationship between the formation of *L. monocytogenes* biofilm and lipid metabolism has not been reported yet. It was worth mentioning that phospholipids were the main source of choline, which could generate acetylcholine under the action of acetylase. Based on the relationship between phospholipids and acetylcholine, as well as their crucial roles in bacterial cell membranes, their changes affected the formation of *L. monocytogenes* biofilm.

### 3.8. Effect of Plantaricin Q7 on Arginine Biosynthesis and Biofilm Formation of L. monocytogenes

**Figure 8:**
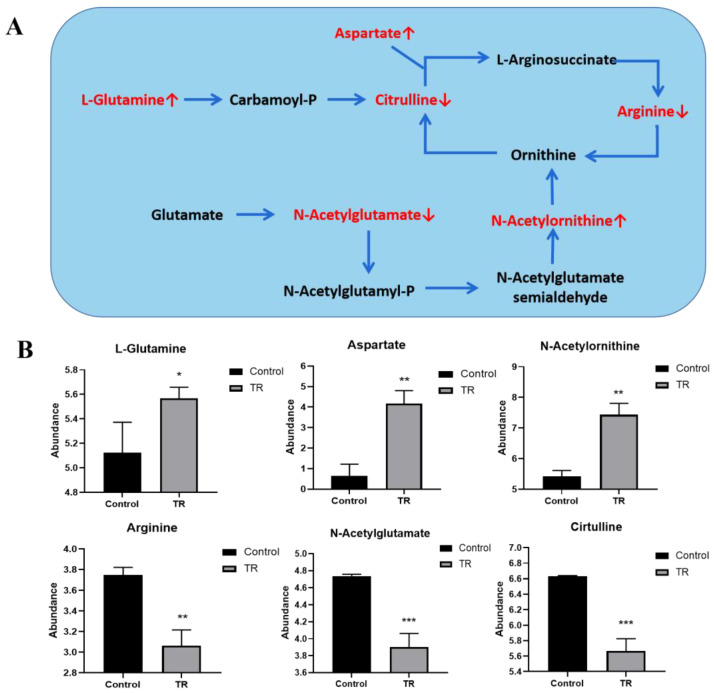
Arginine biosynthesis pathway and major metabolites. (**A**) Arginine biosynthesis pathway; (**B**) effect of plantaricin Q7 on metabolites of arginine biosynthesis pathway in the reduction group. Red indicates metabolites with significant differences. Red ↑ indicates up-regulation and red ↓ indicates down-regulation. * *p* < 0.05, ** *p* < 0.01, *** *p* < 0.001.

It was found that arginine metabolism was significant in the reduction group, and the results are shown in Figure 8A,B. It involved six different metabolites, including L-glutamine, citrulline, aspartate, arginine, N-acetylglutamate and N-acetylornithine. In particular, citrulline, arginine and N-acetylglutamate were down-regulated in the plantaricin Q7 treatment group compared to the control group. Arginine biosynthesis was reported to be critical in the metabolism of various species. The synthesis of extracellular proteases and extracellular amylases was enhanced when arginine synthesis was absent [21,25,30]. Wang et al. [40] found that arginine biosynthesis was particularly active in the early stages of biofilm formation of *B. licheniformis*. Burrell et al. [41] pointed out that spermidine could restore the formation of biofilm of *B. subtilis*, and arginine metabolism was closely related to *B. subtilis* biofilm formation.

Glutamate was reported to be essential for the development of arginine, which was a key compound for cellular metabolism of proline, polyamines, antibiotics, proteins and peptidoglycans [21,40]. As a result, glutamate, as a key substance in arginine biosynthesis, played a decisive role in the formation of *L. monocytogenes* biofilm. Plantaricin Q7 promoted the hydrolysis of extracellular proteins and extracellular polysaccharides of *L. monocytogenes* by down-regulating the synthesis of glutamate, leading to the dissociation of formed biofilms.

### 3.9. Metabolic Pathways of L. monocytogenes Biofilm under Plantaricin Q7 Treatment

**Figure 9:**
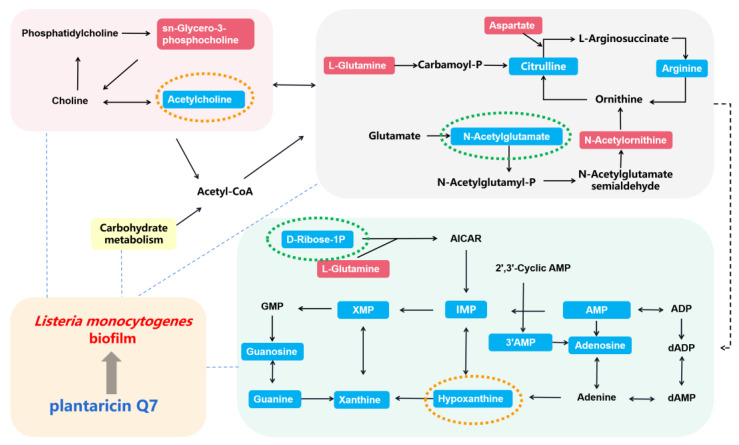
Metabolic pathways related to the inhibition and reduction in *L. monocytogenes* biofilm by plantaricin Q7. The red text boxes indicate up-regulation and the blue text boxes indicate down-regulation. The orange dashed circles represent the two metabolites with the largest difference in fold in the inhibition groups, and the green dashed circles represent the two metabolites with the largest difference in fold in the reduction groups. The remaining dashed lines indicate direct or indirect connections between metabolic pathways. The green background indicates purine metabolism, the gray background indicates arginine biosynthesis, the pink background indicates glycerol phospholipid metabolism, and the light orange background indicates plantaricin Q7 acting on the *L. monocytogenes* biofilm.

According to the results of the above metabolomic analysis, the metabolic pathways related to *L. monocytogenes* biofilm under plantaricin Q7 treatment are summarized in Figure 9. In the inhibition groups, acetylcholine and hypoxanthine (orange dashed circles) showed the largest difference in fold. It was reported that acetylcholine affected the membrane permeability directly, and hypoxanthine was associated with the reduction in bacterial flagellar motility [42,43]. Thus, we concluded that plantaricin Q7 affects the regulation of glycerol phospholipid and purine metabolism by down-regulating the expression of acetylcholine and hypoxanthine, ultimately inhibiting the biofilm formation of *L. monocytogenes*. In the reduction groups, N-acetylglutamate and D-ribose-1-phosphate (green dashed circles) showed the largest difference in fold. It was confirmed that N-acetylglutamate was a key precursor for the synthesis of glutamine, proline, arginine and lysine [21], and D-ribose-1-phosphate was the raw material for purine nucleotide synthesis [44]. Synergism and interaction from metabolism of purine, amino acid, glycerol phospholipid, and arginine biosynthesis integrated the development of *L. monocytogenes* biofilm. The up- and down-regulations of acetylcholine, hypoxanthine, N-acetylglutamate and D-ribose-1-phosphate had important effects on those key pathways. Based on the current results, the specific target of plantaricin Q7 is still unknown, which will be the subject of future research.

## 4. Conclusions

The effects of plantaricin Q7 on *L. monocytogenes* biofilm formation were investigated from the perspective of metabolism through LC-MS/MS analysis. Plantaricin Q7 not only inhibited the synthesis of *L. monocytogenes* biofilm through purine metabolism and glycerol phospholipid metabolism, but also reduced the formed *L. monocytogenes* biofilm by purine metabolism and arginine biosynthesis. Purine metabolism and amino acid metabolism were found to be the common metabolic pathway for plantaricin Q7 to inhibit and reduce *L. monocytogenes* biofilm, which could serve as potential targets to control *L. monocytogenes* biofilm. These findings contributed to the further development of plantaricin Q7 as *L. monocytogenes* biofilm inhibitor to reduce the risk of *L. monocytogenes* contamination in food industry.

## Data Availability

The original contributions presented in the study are included in the article/Supplementary Material, further inquiries can be directed to the corresponding author.

## Supplementary Materials

The following supporting information can be downloaded at: https://www.mdpi.com/article/10.3390/foods13162573/s1, Figure S1: The MIC of nisin; Figure S2: The MIC of plantaricin Q7; Figure S3: Total ion chromatograms of inhibition group and reduction group in positive-ion mode; Figure S4: Scatter plot of PLS-DA model scores of samples in inhibition and reduction group; Figure S5: Enrichment analysis bar chart of KEGG metabolic pathway in samples from inhibition group and reduction group; Table S1: Mobile phase elution gradient; Table S2: The MIC of nisin; Table S3: The MIC of plantaricin Q7; Table S4: The MBC of plantaricin Q7; Table S5: The MBEC of plantaricin Q7.
