# Supplementary material for: Metabolomic Approaches to Study the Potential Inhibitory Effects of Plantaricin Q7 against Listeria monocytogenes Biofilm
Source: Foods. 2024 Aug 17;13(16):2573. doi: 10.3390/foods13162573 (PMC11353926; doi:10.3390/foods13162573)
Supplement: Supplementary file 1 [file foods-13-02573-s001.zip › foods-3153224-Supplementary materials.pdf]

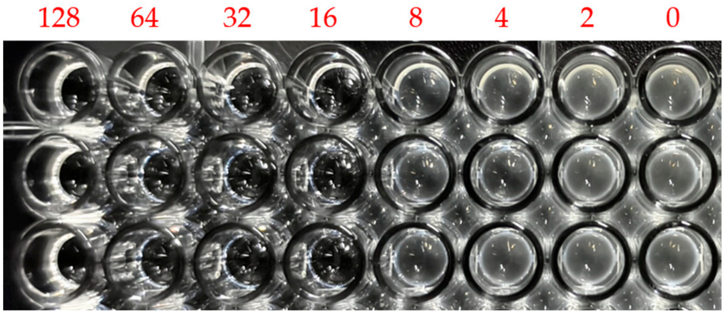

Figure S1. The MIC of nisin on *L. monocytogenes* (16 μg/mL).

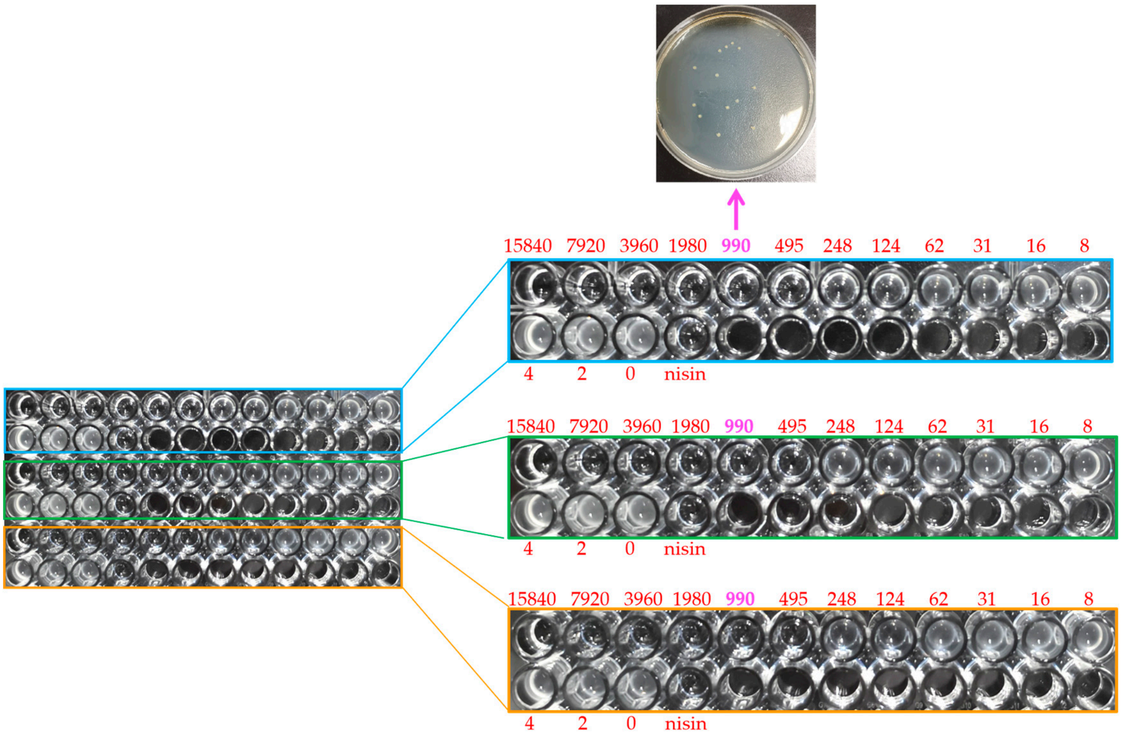

Figure S2. The MIC of plantaricin Q7 on *L. monocytogenes* (495 μg/mL). Three repeated experiments were indicated by blue, green, and orange boxes, respectively. The concentration of plantaricin Q7 in each well was marked in red font with units of μg/mL. At a concentration of 990 μg/mL (magenta font), the antibacterial efficiency of plantaricin Q7 reached 99.9%. The result of undiluted bacterial solution in the corresponding well was displayed at the top.

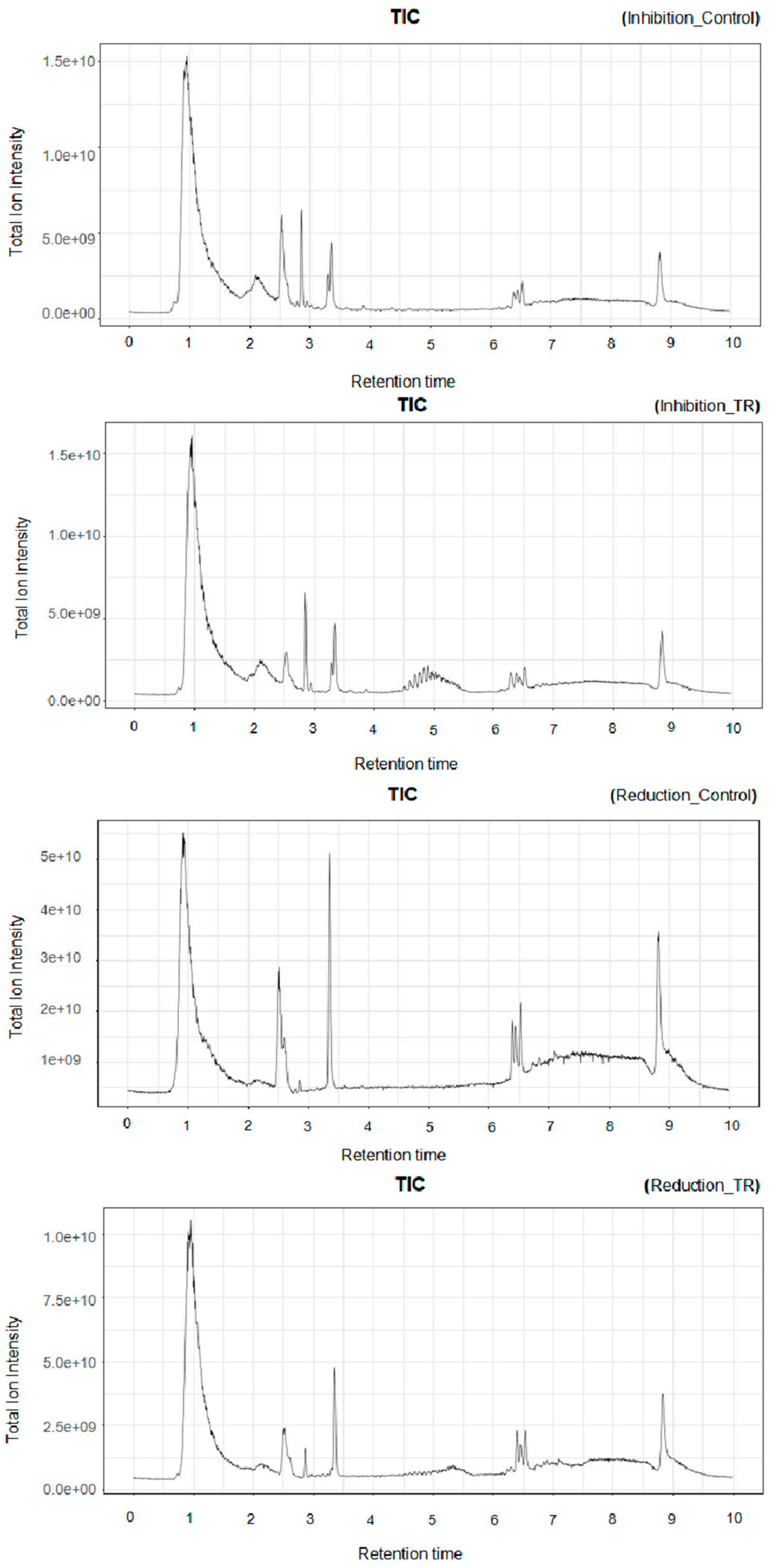

Figure S3. Total ion chromatograms of inhibition group and reduction group in positive-ion mode.

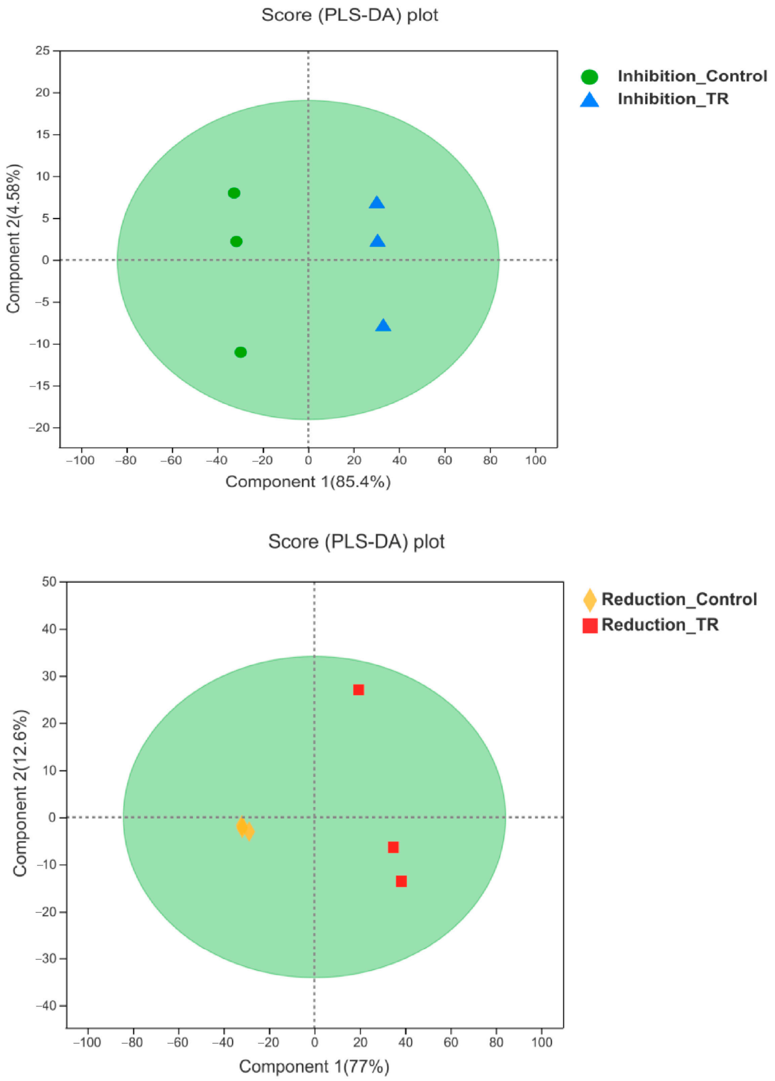

Figure S4. Scatter plot of PLS-DA model scores of samples in inhibition and reduction group.

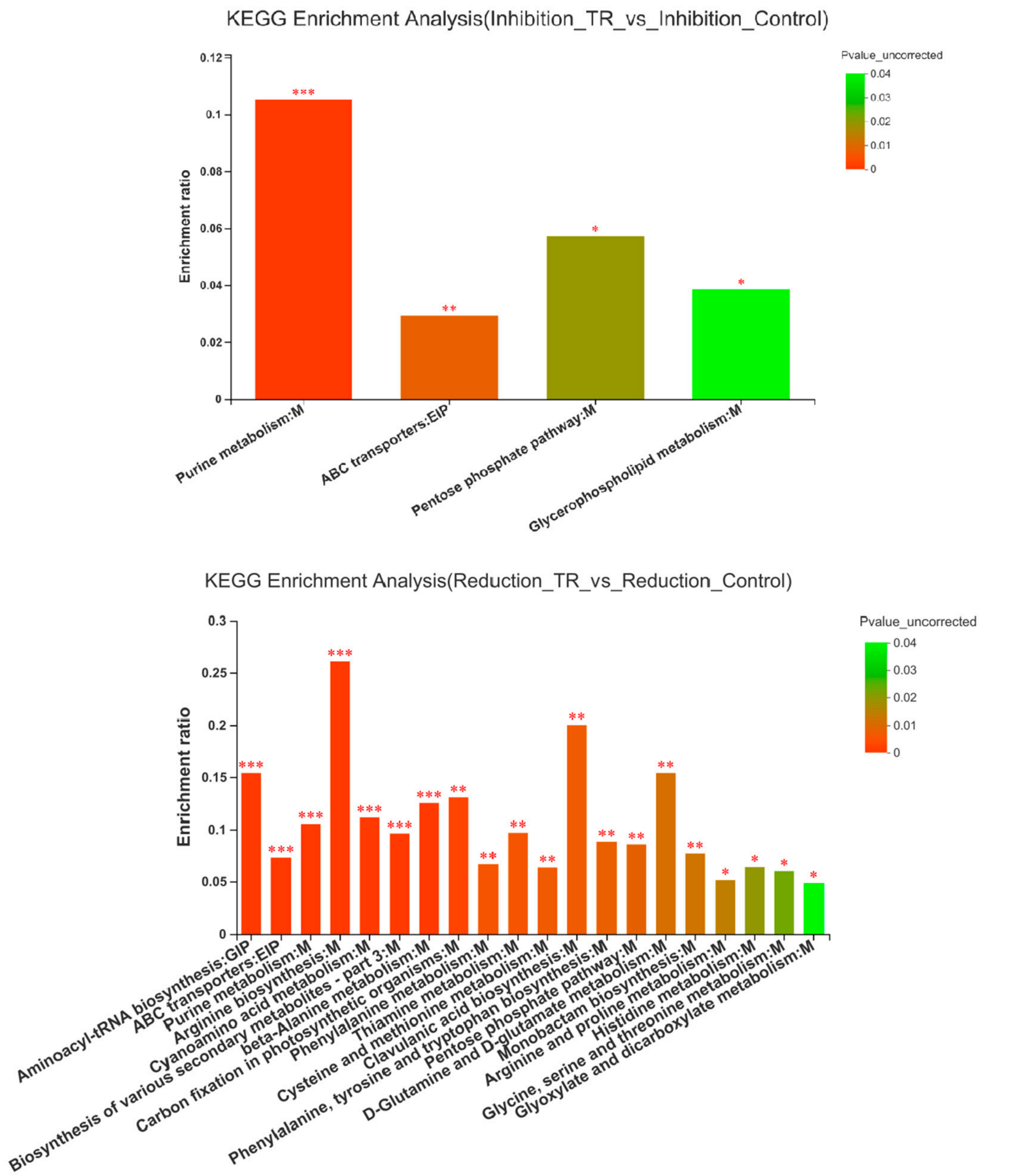

Figure S5. Enrichment analysis bar chart of KEGG metabolic pathway in samples from inhibition group and reduction group.

Table S1. Mobile phase elution gradien.

Time (min)	Flow rate (mL/min)	A (%)	B (%)
0	0.4	100	0
3.5	0.4	75.5	24.5
5	0.4	35	65
5.5	0.4	0	100
7.4	0.6	0	100
7.6	0.6	48.5	51.5
7.8	0.5	100	0
9	0.4	100	0
10	0.4	100	0

Table S2. The MIC of nisin on *L. monocytogenes.*

Nisin (μg/mL)	128	64	32	16	8	4	2	0
1 (OD_{600nm})	0.067	0.065	0.067	0.065	0.152	0.229	0.308	0.344
2 (OD_{600nm})	0.058	0.059	0.056	0.057	0.167	0.174	0.298	0.328
3 (OD_{600nm})	0.059	0.059	0.058	0.058	0.160	0.236	0.281	0.289

Table S3. The MIC of plantaricin Q7 on *L. monocytogenes*.

Plantaricin Q7 (μg/mL)	15840	7920	3960	1980	990	495	248	124
1 (OD_{600nm})	0.057	0.056	0.057	0.063	0.061	0.072	0.134	0.200
2 (OD_{600nm})	0.056	0.059	0.062	0.057	0.060	0.067	0.056	0.185
3 (OD_{600nm})	0.057	0.060	0.068	0.061	0.066	0.078	0.147	0.194
Plantaricin Q7 (μg/mL)	62	31	16	8	4	2	0	Nisin
1 (OD_{600nm})	0.265	0.308	0.381	0.387	0.357	0.382	0.380	0.056
2 (OD_{600nm})	0.232	0.262	0.294	0.359	0.371	0.359	0.368	0.063
3 (OD_{600nm})	0.259	0.271	0.345	0.363	0.343	0.378	0.377	0.057

Table S4. The MBC of plantaricin Q7 on *L. monocytogenes*.

Plantaricin Q7 (μg/mL)	495	990	1980
Viable count (lg CFU /mL)	3.775 ± 0.033^{a}	1.580 ± 0.026^{b}	0.563 ± 0.017^{c}

Note: At a concentration of 990 μg/mL, the bactericidal efficiency of plantaricin Q7 reached 99.9%, defined as its MBC.

Table S5. The MBEC of plantaricin Q7 on *L. monocytogenes*.

Plantaricin Q7 (μg/mL)	1980	3960	7920
Viable count (lg CFU /mL)	3.410 ± 0.098^{a}	1.326 ± 0.051^{b}	0^{c}

Note: At a concentration of 7920 μg/mL, plantaricin Q7 prevented bacterial regeneration in biofilm, defined as its MBEC.
